# Monitoring of Pre-Load on Rock Bolt Using Piezoceramic-Transducer Enabled Time Reversal Method

**DOI:** 10.3390/s17112467

**Published:** 2017-10-27

**Authors:** Linsheng Huo, Bo Wang, Dongdong Chen, Gangbing Song

**Affiliations:** 1 State Key Laboratory of Coastal and Offshore Engineering, Dalian University of Technology, Dalian 116024, Liaoning, China; lshuo@dlut.edu.cn (L.H.); chendongdlut@mail.dlut.edu.cn (D.C.); 2 Key Laboratory of Transportation Tunnel Engineering, Ministry of Education, Southwest Jiaotong University, Chengdu 610031, Sichuan, China; 3 Smart Material and Structure Laboratory, Department of Mechanical Engineering, University of Houston, Houston, TX 77204, USA

**Keywords:** piezoceramic transducer, lead zirconate titanate (PZT), rock bolt, pre-load of a rock bolt, time reversal technique, pre-load monitoring

## Abstract

Rock bolts ensure structural stability for tunnels and many other underground structures. The pre-load on a rock bolt plays an important role in the structural reinforcement and it is vital to monitor the pre-load status of rock bolts. In this paper, a rock bolt pre-load monitoring method based on the piezoceramic enabled time reversal method is proposed. A lead zirconate titanate (PZT) patch transducer, which works as an actuator to generate stress waves, is bonded onto the anchor plate of the rock bolt. A smart washer, which is fabricated by sandwiching a PZT patch between two metal rings, is installed between the hex nut and the anchor plate along the rock bolt. The smart washer functions as a sensor to detect the stress wave. With the increase of the pre-load values on the rock bolt, the effective contact surface area between the smart washer and the anchor plate, benefiting the stress wave propagation crossing the contact surface. With the help of time reversal technique, experimental results reveal that the magnitude of focused signal clearly increases with the increase of the pre-load on a rock bolt before the saturation which happens beyond a relatively high value of the pre-load. The proposed method provides an innovative and real time means to monitor the pre-load level of a rock bolt. By employing this method, the pre-load degradation process on a rock bolt can be clearly monitored. Please note that, currently, the proposed method applies to only new rock bolts, on which it is possible to install the PZT smart washer.

## 1. Introduction

Rock bolts are widely used to stabilize rock masses in tunneling and underground construction [1,2]. However, rock bolt related accidents still happen [3,4] and people’s lives were taken because of the rock bolt failure caused tunnel collapse [5]. Many factors, such as insufficient length, improper grouting, corrosion, and insufficient pre-load, contribute to rock bolt failures [6,7,8,9]. The pre-load on a rock bolt plays an important role in stabilizing rock masses. The reduction or loss of the pre-load of a rock bolt significantly reduces its capacity to stabilize the rock masses. Therefore, the rock bolt monitoring in general and the pre-load monitoring in particular are of great importance [10,11].

Pre-load monitoring of rock bolts receives much attention and employs various techniques and sensors, including vibrating wire load cells [3], embedded strain gauges [12], Fiber Bragg Grating strain sensors [13,14], Brilloun Optical Time Domain Analysis (BOTDA) distributed strain sensors [15], and ultrasonic transducers [16]. Due to their multi-functionalities, including grouting quality monitoring [17,18,19] and delamination detection [20], ultrasonic transducers have been widely researched for monitoring of rock bolts [21,22,23]. Thanks to its advantage of high sensitivity, fast response, wide bandwidth, and dual sensing and actuation function, piezoceramic materials have been widely used as ultrasonic transducers [24]. In particular, the lead zirconate titanate—also known as PZT, a piezoceramic material—is commonly adopted to build ultrasonic transducers due to its strong piezoelectric effect [25]. In addition, PZT transducers have the energy harvesting capacity [26,27,28]. PZT based ultrasonic transducers are commonly used in structural health monitoring and damage detection [29,30,31,32]. 

In recent years, the PZT enabled active sensing approach has been developed to monitor pre-load on bolted connections [33,34]. Based on the fact that an increased pre-load increases the contact area on the contact interface, the wave energy increases before reaching a saturation when the propagated stress across the interface. The applied torque on bolts may change the interfacial characteristics such as stiffness, damping, and true contact area. Once the interfacial characteristics changes are obtained, the tightness of the bolted connections can be determined [35]. A PZT based active sensing method was developed by Wang et al. [36]. In this study, the experimental results shown that the wave energy propagated across the interface is proportional to the torque level before the saturation. Similar results were obtained in the investigation of Huo et al. by using a pair of PZT based smart washers [37]. In addition, the piezoceramic based impedance approach was used for rock bolt pre-stress monitoring [38].

In this paper, a PZT enabled reversal method is proposed to monitor the pre-load of a rock bolt based on stress wave propagation. A PZT transducer, which can function as a sensor and an actuator, is a suitable candidate as a time reversal mirror. The proposed method intends to detect the pre-load status of a rock bolt though ultrasonic wave, which travels between the anchor plate and the bolt shaft. A PZT patch transducer is bonded onto the anchor plate to function as an actuator to generate ultrasonic waves. A smart washer with a sandwiched PZT transducer is installed on the bolt shaft between the anchor plate and the hex nut works as a sensor to detect the propagated stress wave. By analyzing the data collected from the PZT patch and the smart washer through the time reversal technique, different pre-load levels can be detected. Experimental results also demonstrate that it is a simple and feasible approach to monitor the pre-loading level.

## 2. Principle of Rock Bolt Pre-Load Monitoring Using PZT Enabled Time Reversal Method

**Figure 1:**
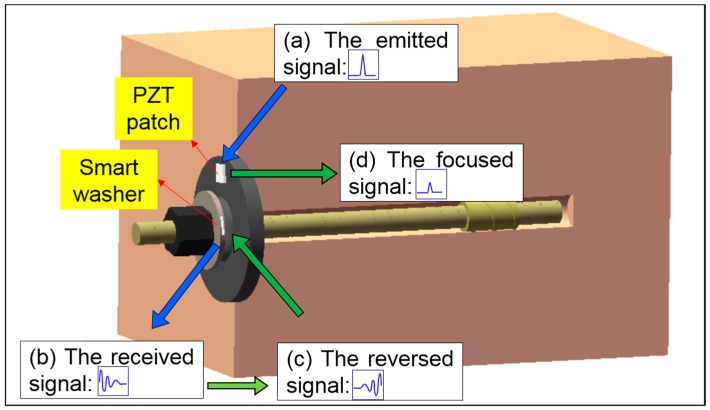
The time reversal pre-load monitoring a rock bolt.

Time reversal is a time domain reverse operation of the received signals and has two properties, namely the temporal focusing and the spatial focusing [39]. In addition, the time reversal technique is suitable for the complicated medium as long as the reciprocity principle holds. Figure 1 shows the assembly of an installed rock bolt. Attached on the anchor plate is a PZT patch, which is used as an actuator in this research to emit a stress wave. On the other hand, a PZT transducer, called a smart washer that is fabricated by sandwiching a PZT patch by a pair of washers [37], is installed between the hex nut and the anchor plate on the rock bolt. The smart washer functions as a sensor to detect the propagated stress waves, which cross the contact surfaces whose effective contact area is influenced by the pre-load on the bolt.

**Figure 2:**
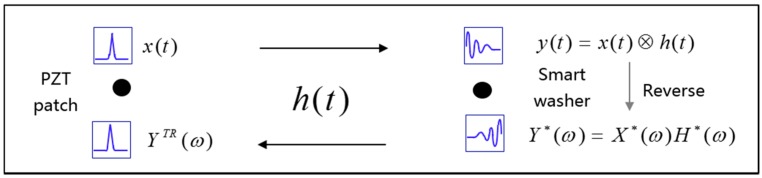
The schematic of time reversal method for active sensing.

A PZT transducer, which can function as a sensor and an actuator, is a suitable candidate as a time reversal mirror in the time reversal process. As shown in Figure 1 and also illustrated in Figure 2, in the time reversal process, the PZT patch emits a stress wave, which propagates along the anchor plate, crossing the contact interface between the smart washer and the anchor plate. The stress wave is then detected by the smart washer. The detected signal is time-reversed and re-emitted by the smart washer. The re-emitted stress wave will be focused at the PZT patch. 

The PZT patch works as an actuator which emits a pulse signal *x*(*t*). The transfer function of the system is assumed as *h*(*t*), and the sensor (smart washer) will detect the received signal which is represented as *y*(*t*). The received signal *y*(*t*) can be expressed as [40]

(1) y(t)=x(t)⊗h(t)

where ⊗ represent the convolution operation. In Equation (1), *y*(*t*) is in time domain, and *Y* (*ω*) is its counterpart in the frequency domain by applying Fourier Transforms which is shown in Equation (2).

(2) Y(ω)=X(ω)H(ω)

where *ω* denotes the angular frequency, and the time reversed signal *y*(−*t*) is equal to the complex conjugated of *Y*(*ω*), as shown in Equation (3),

(3) $$Y^{*}(\omega )=X^{*}(\omega )H^{*}(\omega )$$

Y*(*ω*), X*(*ω*), H*(*ω*) are complex conjugation of *Y*(*ω*), *X*(*ω*), and *H*(*ω*). In the time domain, the emitted signal *x*(*t*) is designed to be symmetric, hence, *X*(*ω*) = X*(*ω*). The focused signal of *Y^TR^* can be expressed as

(4) $$Y^{TR}=Y^{*}(\omega )H(\omega )=X^{*}(\omega )H^{*}(\omega )H(\omega )=X(\omega )H^{*}(\omega )H(\omega )=X(\omega ){\left|H(\omega )\right|}^{2}$$

Through taking the inverse Fourier transform of $Y^{TR}$, the time domain $y^{TR}$ can be obtained as

(5) $$y^{TR}=\frac{1}{2\pi }\int_{-\infty }^{+\infty }X(\omega ){\left|H(\omega )\right|}^{2}e^{i\omega t}d\omega =Cx(t)$$

(6) $$x(t)=\frac{1}{2\pi }\int_{-\infty }^{+\infty }X(\omega )e^{i\omega t}d\omega$$

where $C={\left|H(\omega )\right|}^{2}$ and is independent of the angular frequency ω. The focused signal is affected by only the transfer function *h*(*t*), which is influenced by the pre-load on the rock bolt. More detailed information about the time reverse can be found in Liang at al.’s work [39]. The time reverse method has the advantage of spatial focusing property, temporal focusing property [38], and robust anti-noise ability. With this unique advantage, the noise disturbance from rock bolt environment can be minimized.

## 3. Experimental Setup and Procedure

**Figure 3:**
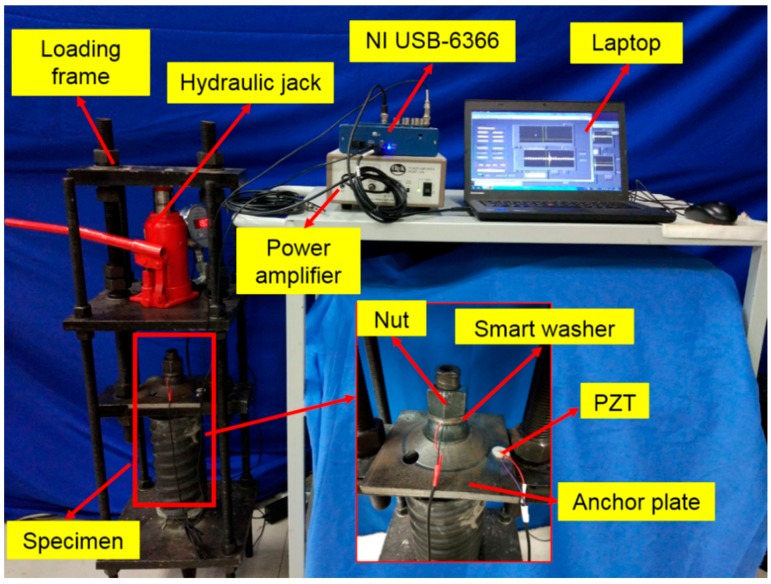
The experimental setup of pre-load monitoring of a rock bolt specimen.

The experimental setup of the rock bolt pre-load monitoring system is illustrated in Figure 3. The rock bolt specimen is installed in a loading frame, which is equipped with a hydraulic jack. With the help of a load cell, the pre-load on the rock bolt can be monitored and controlled. The data acquisition system includes an NI USB-6366 acquisition and control card with 2 MHz maximum sampling rate and a laptop with LabVIEW software program. The power amplifier is used to provide high voltage signal to the drive the PZT devices. 

**Figure 4:**
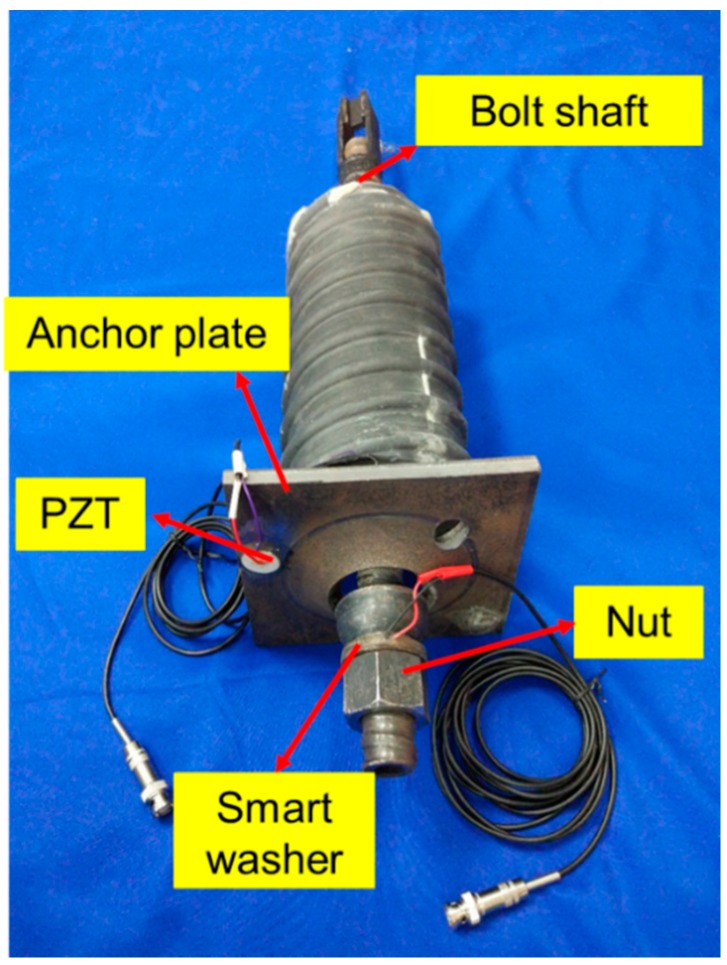
The specimen of smart washer (SW) with connecting wires.

The PZT patch and PZT smart washer along with the rock bolt system are shown in Figure 4. The smart washer is installed between a hex nut and the anchor plate. The PZT patch transducer is bonded onto the anchor plate. The stress wave emitted by the PZT patch propagates through the interfaces between the anchor plate and the nut, and then the propagating stress wave is detected by the smart washer. Please note that the proposed method applies to only new rock bolts, where it is possible to install the PZT smart washer.

In the experiment, the pre-load was applied by the hydraulic jack. The loading procedure was decreased from 54 MPa to zero MPa with a 3-MPa interval, resulting 21 different pre-loading cases. Each pre-loading case was conducted 10 times.

## 4. Experimental Results

**Figure 5:**
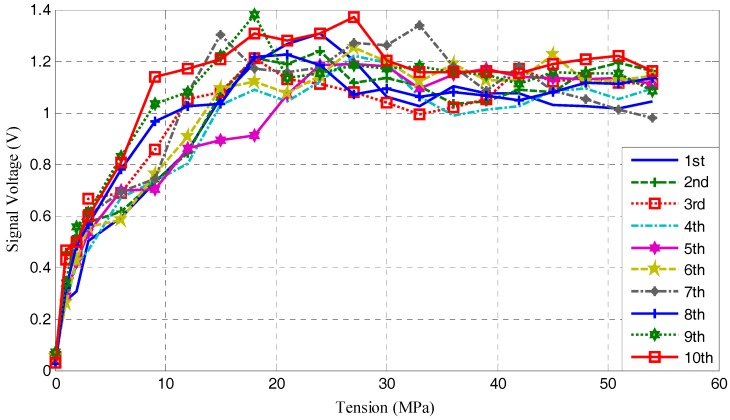
The signal voltages acquired from smart washer.

The experiments were performed, following the procedures described in Section 3. The pre-load values, the average voltage values of the focused signals, and the associated standard deviations are shown in Table 1 and Table 2. The voltages acquired from the smart washer corresponding to Table 1 and Table 2 are plotted in Figure 5.

From Table 1, Table 2, and Figure 5, the amplitude of focused signal barely changed when the pre-load is decreased from 54 MPa to 21 MPa, which means that this is a saturation period. The standard deviations in this period are also very small (<10%). In the range of 21 MPa to 0 MPa, with the further loss of the pre-load, the curves clearly show that there is a decline of the amplitude of the focused signals. This segment is very important for rock bolt pre-load monitoring, since the sharp decrease of amplitude indicates the significant reduction of the pre-load on the rock bolt. 

**Figure 6:**
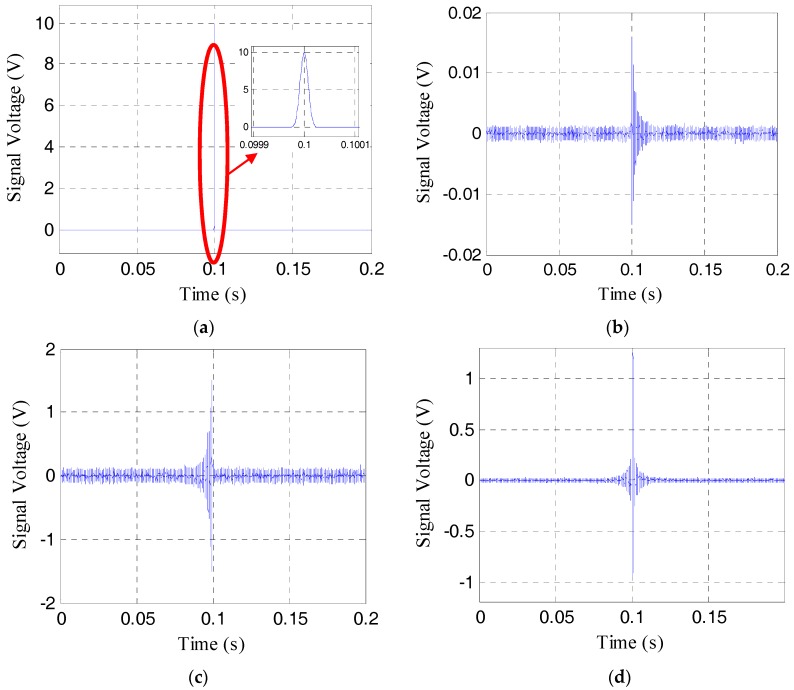
Four different signals in time reverse method at the pre-load of 21 MPa. (**a**) The emitted signal; (**b**) the received signal; (**c**) the reversed signal; (**d**) the focused signal.

**Figure 7:**
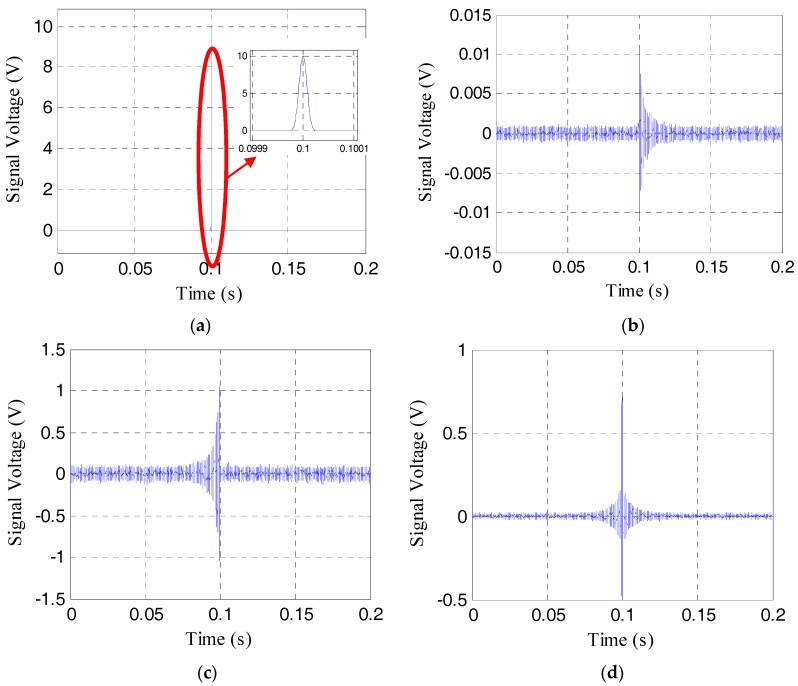
Four different signals in time reverse method at the pre-load of 9 MPa. (**a**) The emitted signal; (**b**) the received signal; (**c**) the reversed signal; (**d**) the focused signal.

**Figure 8:**
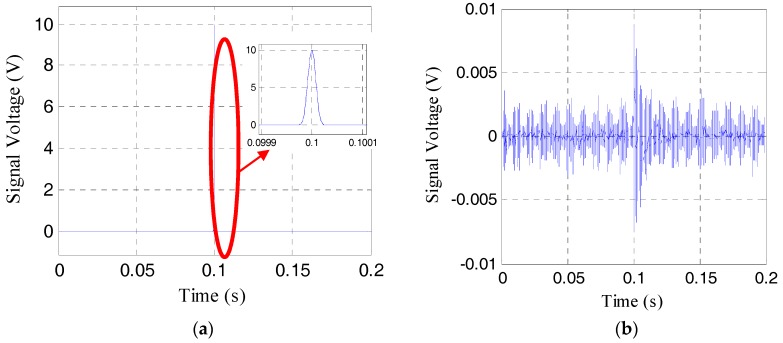

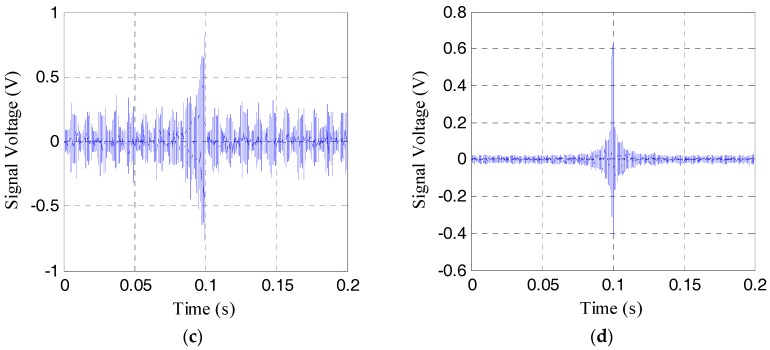
Four different signals in time reverse method at the pre-load of 3 MPa. (**a**) The emitted signal; (**b**) the received signal; (**c**) the reversed signal; (**d**) the focused signal.

**Figure 9:**
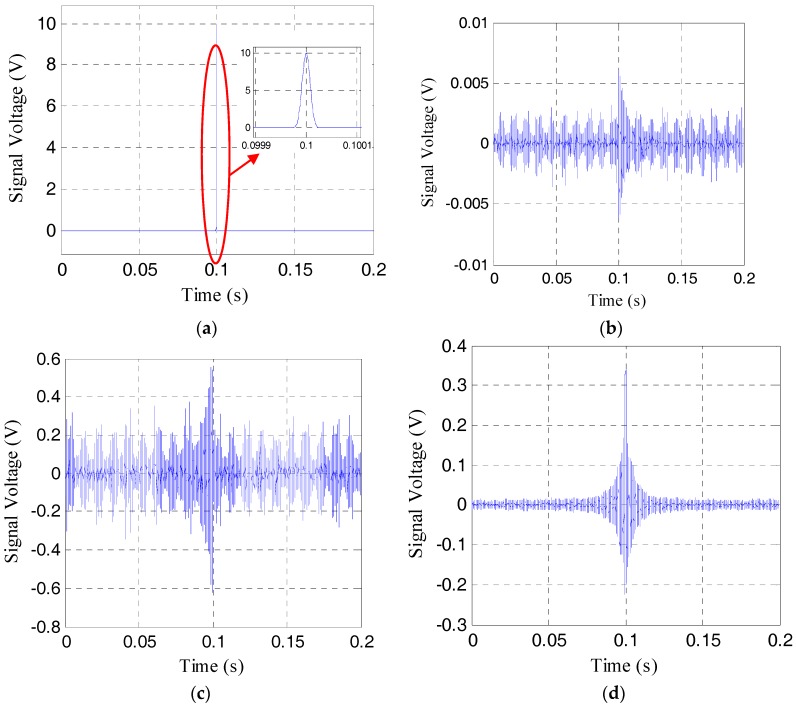
Four different signals in time reverse method at the pre-load of 1 MPa. (**a**) The emitted signal; (**b**) the received signal; (**c**) the reversed signal; (**d**) the focused signal.

To show the effective pre-load monitoring of the rock bolt, especially near the range of the total loss of the pre-load, the results the time-reversal method for the pre-load cases of 21 MPa, 9 MPa, 3 MPa, and 1 MPa are respectively shown in Figure 6, Figure 7, Figure 8 and Figure 9. The four sub-plots with sub-titles (a), (b), (c), and (d) in each figure case are the emitted signals, the received signals, the time-reversed signals and the focused signals, respectively. It should be noticed that the amplitude of the time-reversed signal was magnified 100 times by the LabVIEW data acquisition and control system, as shown in the sub-plots (c), and then re-emitted.

As shown in Figure 6b and Figure 7b, the amplitude of the received signal is more than 0.5 V, which is much larger than the amplitude of noise. The focused signals with the time reverse technique in Figure 6d and Figure 7d can be clearly seen. With the further loss of the pre-load, the amplitude of the received signal becomes much less, as shown in Figure 8b and Figure 9b. In these situations, the focused signal in Figure 8d or Figure 9d, as a result of the time reversal technique, shows a clear peak and enjoys higher signal-to-noise ratio (SNR), which will help to decide the severity of rock bolt pre-load loss. 

As shown in Figure 5, 21 different loading cases in the range of 54 MPa to 0 MPa were investigated. Ten repeated experiments were performed. There are relatively small discrepancies among the repeating experiments. The overall trend of the experimental results, as shown in Figure 5, is clear. During the process of losing pre-load on the rock bolt, in the range of 54 MPa to 21MPa, which is the saturation range, the magnitude of the focused signal changes little with the pre-load reduction. In the range of 20 MPa to 0 MPa, the magnitude of focused signal reduces with the reduction of the pre-load on the rock bolt. In this range, the magnitude of the focused signal can clearly help us to monitor the pre-load on the rock bolt and to detect the total loss of the pre-load. 

## 5. Discussion

**Figure 10:**
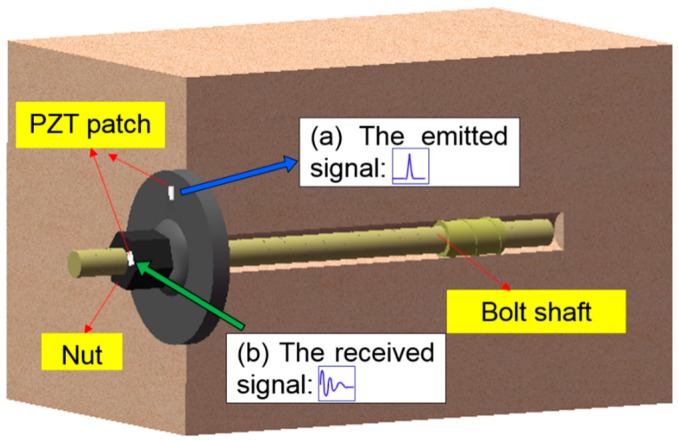
A PZT patch as a sensor on the nut for an existing rock bolt.

Since the proposed method involves installation of the smart bolt along the loading path, this method applies to only new rock bolts. Shown in Figure 10 is a possible new method that can be applied to an existing rock bolt. Please note that a PZT patch, as a sensor, is mounted on the nut, and there is no need of the PZT washer. The location for the PZT patch actuator remains the same. The pre-load on the rock bolt changes the contact area between the nut and the anchor plate, and therefore influences the wave propagation from the PZT actuator to the PZT sensor, based on which the pre-load can be monitored. Another possible location for the PZT sensor is the open end of the rock bolt. In the future, we will experimentally explore this method that can be applied to pre-load monitoring of already-installed rock bolts.

## 6. Conclusions

In this paper, a piezoceramic transducer enabled time reversal method is proposed to monitor the pre-load of a rock bolt. The lead zirconate titanate (PZT) type of piezoceramic material is adopted in this research. A PZT patch transducer bonded on the anchor plate of the rock bolt works as an actuator to generate stress waves. A smart washer with sandwiched PZT patch is installed as a sensor between the hex nut and the anchor plate along the rock bolt to detect the stress wave. With the help of the time reversal technique, it is found through experiments that the magnitude of focused signal clearly decreases with the reduction of the pre-load on a rock bolt before the saturation, which refers to the phenomenon of the small changes of the magnitude of the focused signal with the changes of the pre-load. The proposed method provides an innovative and real time means to monitor the pre-load level of a rock bolt. By employing this method, the pre-load degradation process and the total loss of the pre-load on a rock bolt can be clearly monitored. Future work will study the saturation phenomenon to develop a method to increase the pre-load value when the saturation happens. Future work will also involve the integration of the mobile enabled remote technology [41,42] with the proposed method for wireless rock bolt pre-load monitoring. Since the proposed method applies to only new rock bolts, on which it is possible to install the PZT smart washer. In the future, we will develop a new PZT transducer placement scheme so that the developed method can be extended to pre-load monitoring of already-installed rock bolts.

## Figures and Tables

**Table 1:** The average and standard deviation of the focused signal (V) with pre-load 0–24 MPa.

Pre-Load (MPa)	0	1	2	3	6	9	12	15	18	21	24
Average value (V)	0.0495	0.3412	0.4586	0.5673	0.6964	0.8397	0.9669	1.0986	1.1807	1.1568	1.1919
Standard deviation (V)	0.0148	0.0761	0.0675	0.0541	0.0817	0.1454	0.1207	0.1105	0.1201	0.0774	0.0668

**Table 2:** The average and standard deviation of the focused signal (V) with pre-load 27–54 MPa.

Pre-Load (MPa)	27	30	33	36	39	42	45	48	51	54
Average value (V)	1.1954	1.1525	1.1130	1.1056	1.0945	1.1109	1.1184	1.1190	1.1129	1.1015
Standard deviation (V)	0.0875	0.0664	0.0924	0.0680	0.0517	0.0495	0.0558	0.0496	0.0657	0.0529
